# CXCR3 expression on antigen-experienced B cells is systemically dysregulated in type 1 diabetes

**DOI:** 10.1007/s00125-025-06608-y

**Published:** 2025-11-22

**Authors:** Joanne Boldison, Pia Leete, Emma J. Robinson, Wendy Powell, Joanne Davies, Conor McMullan, Sophie L. Walker, Noel G. Morgan, Stephanie J. Hanna, F. Susan Wong

**Affiliations:** 1 Department of Clinical and Biomedical Sciences, University of Exeter, Exeter, UK; 2 Division of Infection and Immunity, Cardiff University School of Medicine, Cardiff, UK

**Keywords:** Antigen-experienced B cells, CXCR3, Lymphocyte migration, Type 1 diabetes

## Abstract

**Aims/hypothesis:**

The chemokine receptor C-X-C chemokine receptor type 3 (CXCR3) is a key chemoattractant molecule that facilitates the migration of activated T cells to the pancreas, leading to beta cell death. In this study, we investigated CXCR3 responses in B cells during type 1 diabetes progression.

**Methods:**

Peripheral blood samples were obtained from individuals with recent-onset and long-duration type 1 diabetes, who were age- and sex-matched to non-diabetic donors. We isolated peripheral blood mononuclear cells (PBMCs) and examined changes in CXCR3 expression on lymphocytes from donors, performing multiparameter flow cytometry and functional cell culture assays. Human post-mortem pancreatic tissue was obtained from the Exeter Archival Diabetes Biobank. Immunofluorescence staining was used to assess CXCR3 expression in pancreatic tissues.

**Results:**

We observed reduced CXCR3 expression on antigen-experienced B cells in individuals with a long duration of type 1 diabetes, although B cells remained responsive to IFNγ. In individuals who were recently diagnosed, IFNγ treatment resulted in increased CXCR3 expression compared with B cells from non-diabetic donors. B cells in pancreases that were recovered post-mortem from young recent-onset donors lacked CXCR3 expression, but co-staining to detect CD8^+^ T cells revealed a CXCR3^+^CD20^+^CD8^+^ T cell population, with their circulating counterpart showing increased CXCR3 expression.

**Conclusions/interpretation:**

We conclude that the CXCR3 response in antigen-experienced B cells is dysregulated during the progression of type 1 diabetes. CXCR3 expression is limited in CD20^+^ B cells in pancreases from recent-onset individuals diagnosed with type 1 diabetes under 7 years of age, but evident on CD8^+^ T cells that express CD20.

**Graphical Abstract:**

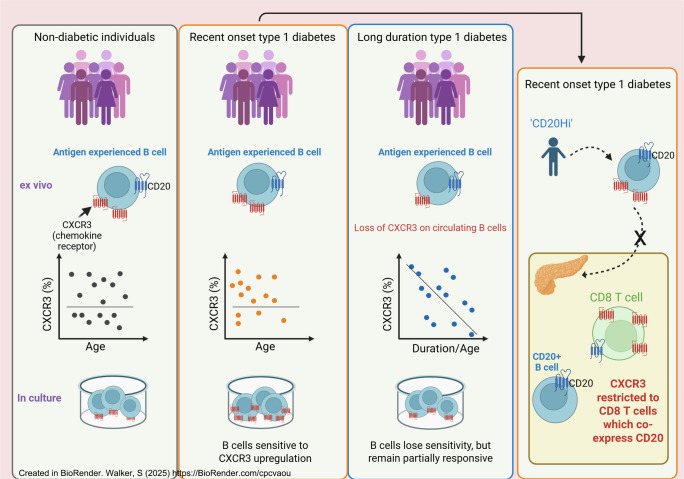

**Supplementary Information:**

The online version of this article (10.1007/s00125-025-06608-y) contains peer-reviewed but unedited supplementary material.


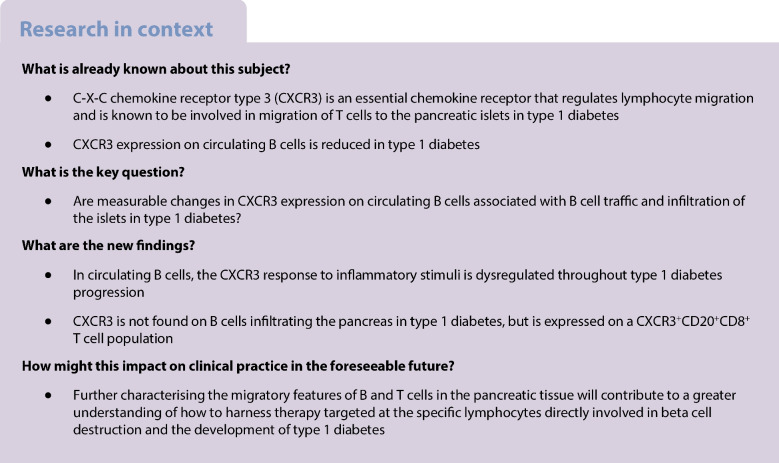


## Introduction

Type 1 diabetes is characterised by immune-mediated beta cell destruction and subsequent insulin deficiency. B cells have multiple functions, including antigen presentation to T cells that results in the production of antibodies, some of which are critical biomarkers for evaluating the future development of type 1 diabetes [1]. B cells have also been identified in pancreatic islets of individuals who died after developing this condition [2]. Pioneering studies have highlighted the existence of possible different phenotypes (or even endotypes) in type 1 diabetes, whereby differing frequencies of CD20^+^ B cells in the insulitic infiltrate in the pancreas are associated with differences in age and extent of residual beta cell mass at diagnosis. Interestingly, higher numbers of islet-associated B cells (CD20^Hi^) equate to an earlier age at diagnosis (often <7 years) and evidence of increased beta cell stress in newly diagnosed individuals [3, 4]. Furthermore, imaging mass cytometry and gene expression studies have linked age at diagnosis, B cell-related genes and differential rates of C-peptide loss [5, 6]. Targeting B cells for depletion using the anti-CD20 antibody rituximab in immunotherapeutic clinical trials in type 1 diabetes temporarily preserved beta cell function, with a decreased rate of C-peptide loss for up to 1 year following treatment [7].

Multiple B cell alterations have been identified in the peripheral blood of individuals diagnosed with type 1 diabetes compared with those who do not have the condition, as summarised recently [8, 9]. These include diminished transitional B cell subsets [10], a loss of anergic, naive (B_ND_, IgD^+^IgM^−^) B cells [11], and B cells that exhibit fewer maturation markers [12], although these findings were not always reproduced in every cohort studied [13]. We previously showed that expression of C-X-C chemokine receptor type 3 (CXCR3), a chemokine receptor that is widely expressed on T cells and on antigen-experienced B cells, is notably reduced on peripheral blood lymphocytes from individuals with long-duration (LD) type 1 diabetes [14]. Furthermore, the chemokines CXCL10 and CXCL11 (ligands for CXCR3) are elevated in the serum of individuals with type 1 diabetes [14]. Both CXCR3 and its ligands have a key role in the pathogenesis of type 1 diabetes, playing a fundamental role in the recruitment of T cells to pancreatic islets [15, 16]. Additionally, CXCR3 and CXCL10 are expressed in the inflammatory infiltrates of individuals with recent-onset (RO) diabetes [17] although B cells expressing CXCR3 have been little studied. CXCR3 is expressed in memory (IgD^−^CD27^+^) and double-negative (DN, IgD^−^CD27^−^, atypical) B cells, and specifically in the CD11c^+^ DN2 B cell subpopulation of DN cells [18]. These CXCR3-expressing B cell subsets give rise to antibody-secreting cells. Furthermore, expression of CXCR3 is important for the migration of plasmablasts to inflamed tissues [19], and is preferentially expressed on IgG1-expressing cells [20].

A recent analysis of discordant twins, only one of whom had developed multiple sclerosis, found that the twin who had developed the disease had substantially fewer CXCR3^+^ B cells in the peripheral circulation, which correlated with an increase in CXCL10 in the cerebrospinal fluid [21]. In rheumatoid arthritis, autoreactive B cells are directed against post-translationally modified antigens and express high levels of CXCR3 [22]. In type 1 diabetes, insulin-specific autoreactive B cells have increased CXCR3 expression compared with non-insulin binding B cells, and are enriched in the pancreatic lymph nodes of individuals with type 1 diabetes [23].

We have reported a reduction in CXCR3 on B cells in the peripheral blood of individuals with type 1 diabetes [14]; however, it is not known whether this result occurs because of a global dysregulation of this chemokine pathway under chronic autoinflammatory conditions, and whether CXCR3 B cells migrate to the pancreas. In this study, we investigated the imbalanced CXCR3 expression on B cells in type 1 diabetes.

## Methods

### Participants in PBMC studies

Adults with RO or LD type 1 diabetes were recruited, together with age- and sex-matched non-diabetic (ND) donors (age was matched to ±4 years). Type 1 diabetes was diagnosed according to criteria established by the ADA [10]. Insulin treatment was commenced within 1 month of diagnosis. The time from diagnosis was less than 1 year for RO individuals and more than 3 years for LD individuals. The age- and sex-matched ND individuals were seronegative for islet-specific autoantibodies, with no personal or family history of type 1 diabetes or other autoimmune conditions. Ethnicity data were collected based on self-report; however, they are not included in this study due to very limited diversity in our cohort. Information for all donors is provided in electronic supplementary material (ESM) Table 1. Power calculations were performed based on a one-way ANOVA (fixed effects), with a large effect size of 0.4 (Cohen’s *f*), at 80% power and a significance level of 0.05. Based on this calculation, we required 22 donors in each group; however, only 12 RO individuals were included for ex vivo analysis, and only a minimum of six donors were included in each cohort for functional assays.

### Tissue biobank

Ethical approval was granted by the West of Scotland Research Ethics Service (REC reference 15-WS-0258). Information for the three individuals in the Exeter Archival Diabetes Biobank (EADB) is given in ESM Table 1.

### Peripheral blood samples

Peripheral blood mononuclear cells (PBMCs) were isolated from heparinised samples of whole blood via density gradient centrifugation using Lymphoprep (STEMCELL Technologies, Cambridge, UK), and were stored as aliquots in liquid nitrogen, after cooling overnight to −80°C, at a controlled rate of −1°C/min, in Cryo-Stor freezing medium (STEMCELL Technologies).

### CXCR3 isoform assessment

Naive and memory B cells were isolated from thawed human PBMCs (30–215×10^6^) using the EasySep Human Memory B cell Isolation Kit (STEMCELL Technologies), according to the manufacturer’s protocol. The purity of each subset was determined by flow cytometry (FACSCanto II, BD Biosciences) using the appropriate antibodies (listed in ESM Table 2). RNA was then extracted using a Qiagen RNeasy Plus Mini Kit and RNeasy MinElute Cleanup Kit. Further details are provided in ESM Methods.

### B cell functional assays

B cells were isolated by magnetic activated cell sorting (MACS) using the EasySep Human B cell Isolation Kit (STEMCELL Technologies), according to the manufacturer’s instructions. B cells (2×10^5^) were then cultured with various stimuli, including 0.5 μg/ml CpG (cytidine monophosphate guanosine), 1 μg/ml anti-CD40 and 5 μg/ml anti-IgM (BCR), alone and in combination with 10 ng/ml IFNγ, for 5 days before collection of supernatants and cell harvesting for flow cytometric staining. All cells were cultured with 10% FBS, 1% penicillin–streptomycin and 1% l-glutamine in RPMI.

### Flow cytometry

Single-cell suspensions were incubated with TruStain (anti-human CD16/32; BioLegend) for 10 min at 4°C, followed by fluorochrome-conjugated monoclonal antibodies against T cell surface markers for 30 min at 4°C. Multiparameter flow cytometry was carried out using the monoclonal antibodies listed in ESM Table 2. Dead cells were excluded from analysis using LIVE/DEAD Exclusion Dye (Invitrogen). Cells were acquired using an LSRFortessa cytometer with FACS Diva software (BD Biosciences) or a Cytek Aurora with SpectroFlo software (Cytek Biosciences), and analysis was performed using FlowJo software (BD Biosciences).

### Cytokine measurement

Supernatants were taken from cell culture assays at the 5 day endpoint for CXCL9, CXCL10 and CXCL11 analysis. Cytokines were measured using a flow cytometry-based immunoassay (LEGENDplex, BioLegend), according to the manufacturer’s instructions. Measurement was carried out on a BDCanto using FACS Diva software, and analysis was performed using LEGENDplex software (BioLegend).

### Multiplex immunofluorescence staining

Staining for glucagon, insulin, CD20, CD8 and CXCR3 was performed on 4 µm sections of formalin-fixed paraffin-embedded (FFPE) pancreatic sections, using validated, commercially available primary antibodies (see ESM Table 2), optimised for use with the OPAL 6-plex Manual Detection Kit (Akoya Biosciences, Marlborough, MA, USA; SKU NEL861001KT), according to the manufacturer’s protocol with minor adaptations (see ESM Methods for more details).

### Statistical analysis

Non-parametric tests were favoured throughout our study due to the limited sample size. Where appropriate, and where data were normally distributed, parametric tests were used. Statistical significance was determined by either a non-parametric Mann–Whitney or Wilcoxon matched-pairs signed rank test to measure between two unpaired and paired variables, respectively. When more than two matched variables were compared, a non-parametric Friedman’s test with Dunn’s multiple comparison was used. For multiple comparison testing between stimulations, all conditions were compared with the unstimulated control. A non-parametric Kruskal–Wallis test with Dunn’s multiple comparison was used to measure more than two variables when unrelated. A two-way ANOVA with Tukey’s multiple comparison test was performed when two categorical independent factors were included. The stimulation index was calculated by calculating the fold change over the unstimulated control, and differences between cohorts were tested using a two-way ANOVA. Tukey’s multiple comparison test was used to measure differences between cohorts for each individual stimulation, and means were measured between groups, independent of individual stimulations, to test for differences between cohorts. For the correlation analysis, if data were normally distributed, a Pearson correlation coefficient was calculated, otherwise a Spearman’s correlation was performed. The specific statistical test for each dataset is provided in the figure legends. Statistical analysis was performed using GraphPad Prism (GraphPad Software, San Diego, CA, USA). Conventional α=0.05 type 1 error rates were used to determine statistical significance for all tests.

## Results

### Peripheral CXCR3 B cell upregulation occurs after IFNγ treatment despite a reduction in basal CXCR3 expression ex vivo

**Fig. 1:**
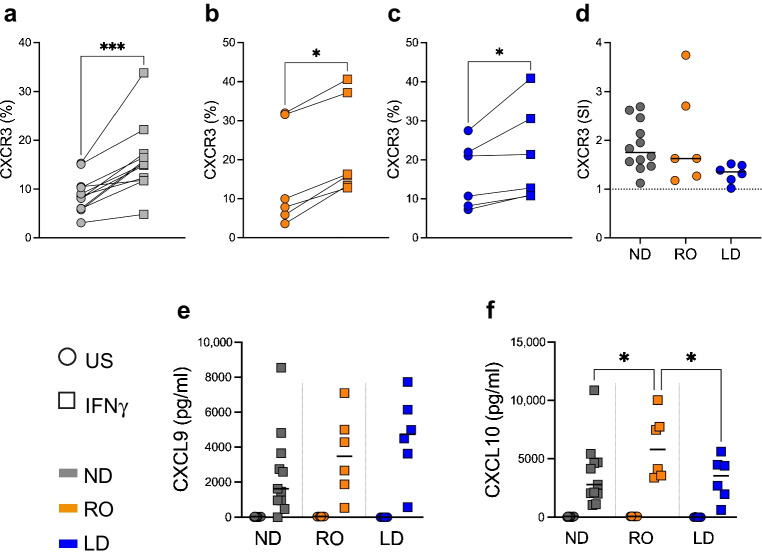
IFNγ upregulated B cell CXCR3 expression ex vivo. CD19^+^ B cells were analysed after a 5 day stimulation with 10 μg/ml IFNγ (squares) or without IFNγ (unstimulated; circles) in three donor cohorts: (**a**) ND (*n*=12, grey), (**b**) RO (*n*=6, orange) and (**c**) LD (*n*=6, blue). Differences were analysed using the Wilcoxon matched-pairs signed rank test. (**d**) CXCR3 stimulation index (fold change over unstimulated control) for each donor cohort. (**e**, **f**) Secreted CXCL9 (**e**) and CXCL10 (**f**) expression levels from supernatants after 5 days of culture. Horizontal lines indicate the median. Differences were analysed using two-way ANOVA with Tukey’s multiple comparison test. Statistically significant differences are indicated using asterisks: *p<0.05, ***p<0.001. SI, stimulation index; US, unstimulated

Previously, we observed a reduction of CXCR3 expression on memory B cells from donors with type 1 diabetes, predominantly in individuals with a diagnosis for more than 3 years [14]. We sought to understand whether B cells from individuals with type 1 diabetes also show an overall reduction in response to IFNγ, an inflammatory cytokine that upregulates CXCR3 expression. We treated isolated B cells with IFNγ for 5 days, and found increased CXCR3 expression compared with unstimulated cultures in ND donors and individuals with type 1 diabetes, whether RO or LD (Fig. 1a–c). Although we observed a statistically non-significant reduction in CXCR3 upregulation in the LD individuals (*p*=0.058) when expression was normalised (by fold change) to the unstimulated control, this cohort had similar secretion levels of the CXCR3 ligand CXCL9 after IFNγ treatment to that in ND donors (Fig. 1e). We did note an increase in CXCL10 secretion from RO donors, compared with ND donors, when treated with IFNγ (Fig. 1f).

### Antigen-experienced B cells from RO donors with type 1 diabetes have greater CXCR3 upregulation in vitro

**Fig. 2:**
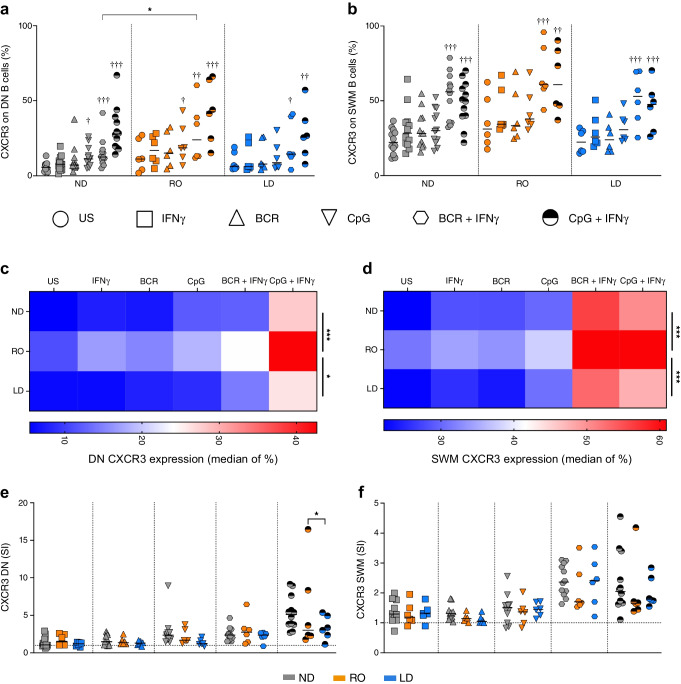
DN and SWM B cells from RO donors increase CXCR3 to a greater extent than those from ND donors. ND donors (*n*=12, grey), RO donors (*n*=6, orange) and LD donors (*n*=6, blue) were assessed for CXCR3 responses to various stimuli as indicated by the symbols. Isolated B cells from PBMCs were cultured for 5 days before flow cytometric measurement, and post-analysis gating was performed to identify DN B cells (**a**, **c**, **e**) and SWM B cells (**b**, **d**, **f**). (**a**, **b**) Percentage CXCR3 expression on B cell subsets. Differences were analysed using a Friedman test with Dunn’s multiple comparison for all stimuli compared with unstimulated control (^†^*p*<0.05, ^††^*p*<0.01, ^†††^*p*<0.001) and a two-way ANOVA with Tukey’s multiple comparison test for differences between cohorts (asterisks indicate statistical significance, *p<0.05). Horizontal lines indicate the median (**c**, **d**) Median expression of CXCR3 by heatmap. A two-way ANOVA with Tukey’s multiple comparison test was used to compare overall cohort differences independent of stimulations (*p<0.05, ***p<0.001). (**e**, **f**) Stimulation index for CXCR3 expression normalised to that of unstimulated cells (fold change, dotted line). Horizontal lines indicate the median. Two-way ANOVA with Tukey’s multiple comparison test was used to test for differences between cohorts: *p<0.05. SI, stimulation index; US, unstimulated

We analysed whether stimulation with IFNγ altered B cell subset frequencies in culture, classified by the expression of IgD and CD27 (ESM Fig. 1a). IFNγ increased DN (IgD^−^CD27^−^) B cells in samples from ND individuals, with a reciprocal decrease in naive B cells (ESM Fig. 1b), but this was less apparent in samples from donors with type 1 diabetes (ESM Fig. 1c). Next, we investigated whether the various B cell subsets had altered functional responses to adaptive B cell receptor (BCR; anti-IgM and anti-CD40) or innate Toll-like receptor (TLR9; CpG) stimulation. Isolated B cells from different cohorts were treated with various stimuli and analysed for CXCR3 expression (Fig. 2) (for gating controls, see ESM Fig. 2a). First, CXCR3 upregulation was limited under the BCR stimulation culture conditions (including in naive B cells that express IgM), indicating that IFNγ is required for a robust CXCR3 response, as addition of this cytokine to cultures increased CXCR3 levels in B cell subsets in all donor groups (Fig. 2 and ESM Fig. 2b, c). Second, both naive (IgD^+^CD27^−^) and unswitched memory (IgD^+^CD27^+^) B cells showed no differences between cohorts in terms of CXCR3 expression under any culture condition (ESM Fig. 2b, c).

In the DN and switched memory (SWM; IgD^−^CD27^+^) subsets, we observed mostly higher expression of CXCR3 in RO individuals compared with ND and LD individuals (Fig. 2a–d). When we assessed the median of CXCR3 expression levels under all conditions, for both DN and SWM B cells (as shown by heatmaps in Fig. 2c and Fig. 2d, respectively), it was evident that CXCR3 expression in RO donors was more upregulated under all culture conditions in comparison with both ND (*p*<0.001) and LD individuals (*p*<0.05). In DN B cells, when expression was normalised to that in the unstimulated control, we observed a decrease in the magnitude of CXCR3 expression in LD donors, in comparison with the RO cohort, in B cells that were stimulated with CpG and IFNγ (Fig. 2e). However, this reduced magnitude of CXCR3 expression in LD individuals was not echoed in the SWM population (Fig. 2f).

**Fig. 3:**
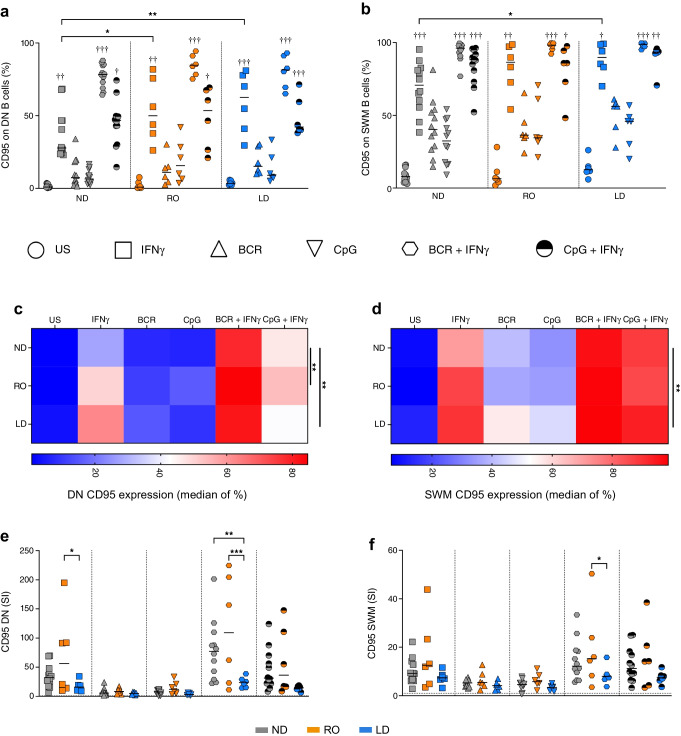
CD95 upregulation in B cells from LD donors activated with IFNγ coupled with a BCR stimulus is more restricted compared with cells from RO donors. ND donors (*n*=12, grey), RO donors (*n*=6, orange) and LD donors (*n*=6, blue) were assessed for CD95 responses to various activation stimuli as indicated by the symbols. Isolated B cells from PBMCs were cultured for 5 days before flow cytometric measurement, and post-analysis gating was performed to identify DN B cells (**a**, **c**, **e**) and SWM B cells (**b**, **d**, **f**). (**a**, **b**) Percentage CD95 expression on B cell subsets. Differences were analysed using a Friedman test with Dunn’s multiple comparison for all stimuli compared with unstimulated control (^†^*p*<0.05, ^††^*p*<0.01, ^†††^*p*<0.001) and a two-way ANOVA with Tukey’s multiple comparison test for differences between cohorts (asterisks indicate statistical significance; *p<0.05, **p<0.01). Horizontal lines indicate the median (**c**, **d**) Median expression of CD95 by heatmap. A two-way ANOVA with Tukey’s multiple comparison test was used to compare overall cohort differences independent of stimulations (**p<0.01). (**e**, **f**) Stimulation index for CD95 expression normalised to that of unstimulated cells (fold change, dotted line). Horizontal lines indicate the median. Two-way ANOVA with Tukey’s multiple comparison test was used to test for differences between cohorts: *p<0.05, **p<0.01, ***p<0.001. SI, stimulation index; US, unstimulated

We also evaluated B cell activation using the surface marker CD95 (Fig. 3) (for gating controls, see ESM Fig. 2d). Such activation was increased when cells were stimulated with IFNγ alone in donors with type 1 diabetes, and this was evident in both DN and SWM B cells in both RO and LD individuals (Fig. 3a, b). However, when stimulated via the innate TLR9 signalling pathway using CpG or the adaptive BCR signalling pathway using anti-CD40/anti-IgM, CD95 expression was similar in all cohorts, even with the addition of IFNγ (Fig. 3a–d). Interestingly, when expression was normalised to that in the unstimulated control, we showed that culturing B cells with CpG or anti-CD40/anti-IgM with additional IFNγ resulted in both the DN and SWM cells having reduced expression of CD95 in LD donors (Fig. 3e, f). We found no differences in CD95 expression on naive or unswitched memory B cell phenotypes (ESM Fig. 2e, f). Altogether our data demonstrate that antigen-experienced (IgD^−^) B cells from RO donors are more sensitive to upregulation of CXCR3 in culture, compared with those from ND, suggesting that B cells at clinical onset are more reactive overall. However, this CXCR3 sensitivity is lost in LD donors, coupled with a more restricted increase in CD95 upon stimulation with IFNγ combined with a TLR9 or a BCR signal.

### Reduction of CXCR3 in memory B cells in type 1 diabetes is not due to a selected CXCR3 isoform

We next wished to clarify whether the reduction of CXCR3 expression on memory B cells observed ex vivo was due to differential CXCR3 isoform expression in donors with type 1 diabetes and donors without diabetes. Data from RO and LD donors were combined for this analysis. We studied the RNA expression of various isoforms of CXCR3 (CXCR3A, CXCR3B and CXCR3alt) in naive and memory B cells. There was no difference in expression of any isoform in naive or memory B cells between individuals with type 1 diabetes compared with the donors without diabetes. Statistically significant differences in expression of all isoforms were observed between naive and memory B cells within each study group (*p*<0.01) (ESM Fig. 3a). To ascertain whether CXCR3 isoform expression changes in response to B cell stimulation, we activated freshly isolated memory B cells from ND donors using CXCL10. There was a reduction in surface expression of CXCR3, measured by flow cytometry, on stimulated memory B cells (*p*<0.01, ESM Fig. 3b). We found no difference in RNA expression of any of the isoforms between unstimulated and stimulated memory B cells (ESM Fig. 3c).

### Reduced CXCR3 expression on B cells is associated with individuals with a long duration of type 1 diabetes

**Fig. 4:**
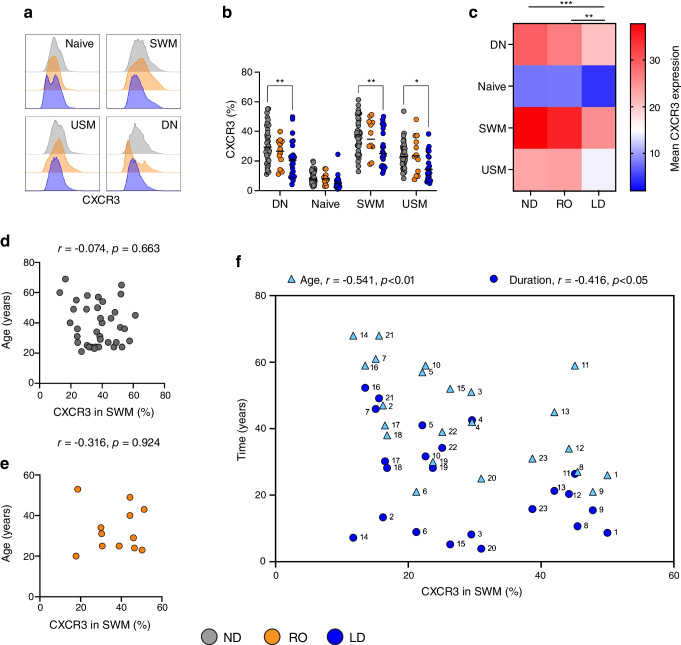
Reduced expression of CXCR3 on B cells correlates with disease duration. CXCR3 expression on B cell subsets from ND donors (*n*=38, grey), RO donors (*n*=12, orange) and LD donors (*n*=23, blue) is shown. Populations were defined by IgD and CD27 gating on the CD19^+^ B cell population: naive (IgD^+^CD27^−^), SWM (IgD^−^CD27^+^), USM (IgD^+^CD27^+^) and DN (IgD^−^CD27^−^). (**a**) Histograms showing CXCR3 expression for each key B cell subset, with donor cohorts overlaid. (**b**) Summary graph of CXCR3 expression. Horizontal lines indicate the median. Differences were analysed using two-way ANOVA with Tukey’s multiple comparison test. (**c**) Heatmap showing overall CXCR3 expression. A two-way ANOVA with Tukey’s multiple comparison test was used to test for overall differences between cohorts, independent of B cell subset. (**d**, **e**) Lack of age correlation in ND donors (**d**) and RO donors (**e**) with CXCR3 expression in SWM B cells. (**f**) Statistically significant correlations between age (blue triangles) and disease duration (blue circles) and CXCR3 expression in SWM B cells in LD donors. Numbers represent the donor code. Pearson correlation coefficient was calculated for associations between ND and LD donors, and Spearman’s correlation was performed for RO associations with ND donors. Statistically significant differences are indicated using asterisks: *p<0.05, **p<0.01, ***p<0.001. USM, unswitched memory

We next sought to identify whether the reduced CXCR3 expression on circulating B cells was associated with age and duration of disease. We first confirmed our previous observations of a reduced expression of CXCR3 on B cell subsets, specifically in donors with LD type 1 diabetes (Fig. 4a–c). We noted a lower expression of CXCR3 in the DN B cell subset (IgD^−^CD27^−^), in comparison with ND donors (*p*<0.01), a feature of this cell type that has not previously been described in type 1 diabetes (Fig. 4b, c). In the ND individuals, we found no relationship between age and the expression of CXCR3 in the SWM or DN B cell populations (Fig. 4d and data not shown), and this was also observed in the RO individuals (Fig. 4e and data not shown). However, in LD donors, age (blue triangles) and duration of disease (blue circles) were associated with a lower expression of CXCR3 on SWM B cells (Fig. 4f). The lower expression of CXCR3 on DN B cells in LD individuals also correlated with age (*r*=−0.406, *p*=0.054) and duration of disease (*r*=0.357, *p*=0.093); however, these correlations were not statistically significant (data not shown). No correlation between CXCR3 expression and age of diagnosis in LD donors was found (data not shown). Overall, these data indicate that the lower expression of CXCR3 on antigen-experienced circulating B cells is associated with a longer duration of disease.

### Pancreatic B cells in donors with RO type 1 diabetes do not express CXCR3

**Fig. 5:**
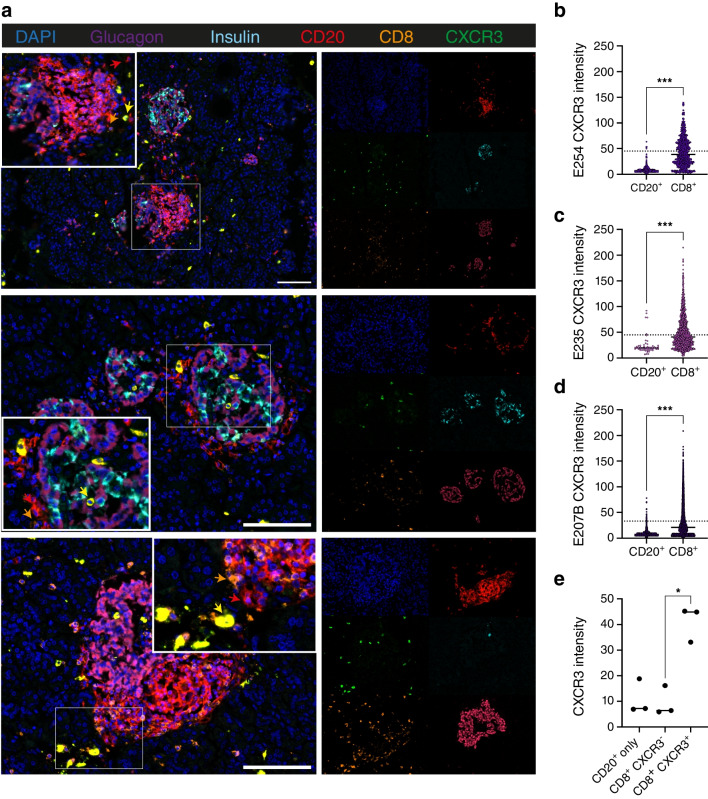
Pancreatic B cells in RO donors express little CXCR3. Pancreatic sections from the EADB were stained using a multiparameter immunofluorescent panel comprising DAPI (blue), anti-glucagon (pink), anti-insulin (aqua), anti-CD20 (red), anti-CD8 (orange) and anti-CXCR3 (green). All donors investigated had young-onset diabetes (diagnosed at age <7 years) and have a high number of immune cell infiltrates. (**a**) Representative images of merged channels (left panel) with zoom islet overlay: arrows indicate double-positive CD8^+^CXCR3^+^ T cells (yellow), CD20^+^ B cells (red) and CD8^+^ T cells (orange). Right images show single fluorescent images for each channel. A representative range of pancreatic islets from two different individuals is shown, both with and without remaining insulin-positive beta cells. Scale bar, 100 μm. (**b**–**d**) Quantification of the maximum intensity of CXCR3 on CD20^+^ B cells and CD8^+^ T cells for donors E254 (**b**), E235 (**c**) and E207B (**d**). The dotted line represents the median of CXCR3 expression on CD8^+^CXCR3^+^ cells. Differences were analysed using a Mann–Whitney test. (**e**) Maximum intensity of CXCR3 expression on CD20^+^ only B cells, CD8^+^CXCR3^−^ cells and CD8^+^CXCR3^+^ cells; the horizontal solid lines represent the median. Differences were analysed using a Friedman test with Dunn’s multiple comparison test. The total number of cells analysed in the whole pancreatic slide per donor was 977,399 for E207B, 635,014 for E235, and 144,824 for E254. Statistically significant differences are indicated using asterisks: *p<0.05, ***p<0.001

We next investigated whether CXCR3 expression was present on the CD20^+^ B cell population found in the pancreas of RO individuals studied post-mortem. Using the EADB, we evaluated three pancreatic donors who had been diagnosed with type 1 diabetes as young children (ESM Table 1) and who were characterised as having an abundant CD20^+^ B cell population [3, 4]. Using multiparameter immunofluorescence techniques, we evaluated CXCR3 expression on CD20^+^ B cells in the pancreases of these individuals (Fig. 5). Representative images from pancreases stained with DAPI and antibodies against insulin, glucagon, CD20, CD8 and CXCR3 are shown in Fig. 5a. We demonstrated that, overall, very few CD20^+^CXCR3^+^ B cells were identified in the tissues, with each donor having a lower fluorescence intensity of CXCR3 on CD20^+^ B cells compared with CD8^+^ T cells (Fig. 5b–d), and showed that the median CXCR3 expression on CD20^+^ B cells was similar to that for CD8^+^CXCR3^−^ T cells (Fig. 5e). We also noted that much of the CXCR3 staining occurred in cells in the surrounding parenchyma rather than the islets, and was not often detected within inflamed islets where large clusters of CD20^+^ B cells could be observed. Furthermore, the CXCR3 staining that we identified in the parenchyma co-localised with strongly positive CD8 staining, therefore suggesting that CXCR3 expression in the pancreas of young-onset donors is restricted to CD8^+^ T cells.

### CD8^+^CXCR3^+^ T cells in the pancreas of donors with RO type 1 diabetes express CD20 and have increased CXCR3 expression

**Fig. 6:**
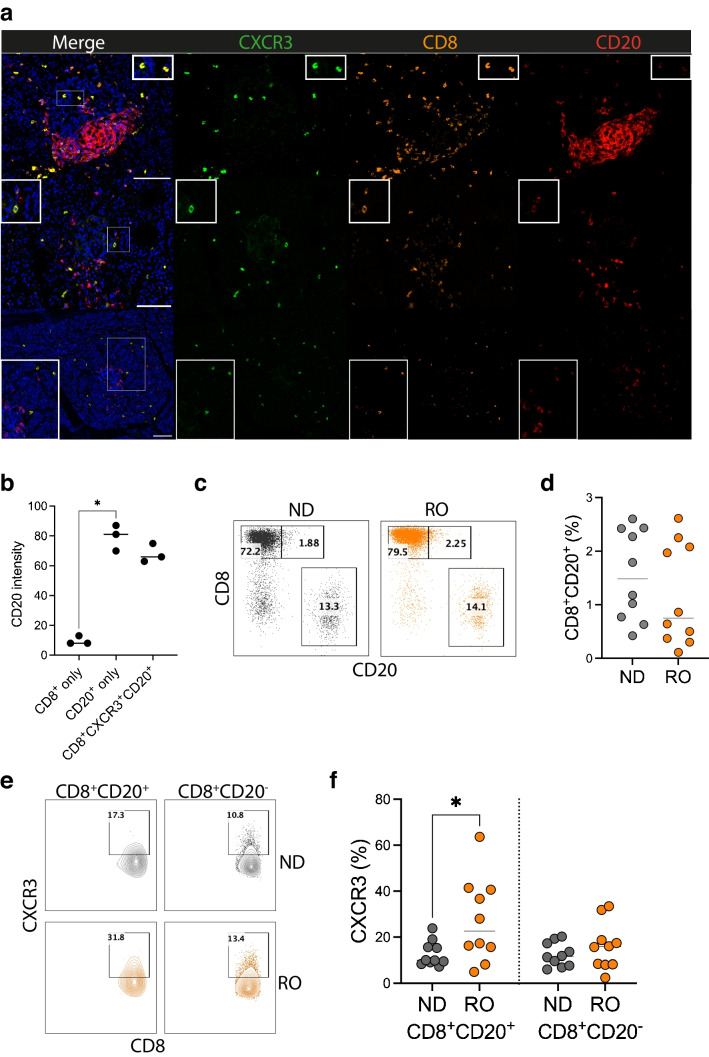
CD8^+^CXCR3^+^ T cells express CD20 in the pancreas of RO donors. (**a**) Pancreatic sections from the EADB were stained using a multiparameter immunofluorescent panel comprising DAPI (blue), anti-CD20 (red), anti-CD8 (orange) and anti-CXCR3 (green). White boxes indicate examples of CXCR3^+^ CD8^+^ T cells expressing CD20. Scale bars, 100 μm. (**b**) Quantification of the maximum intensity of CD20 expression from each donor for CD8^+^ only, CD20^+^ only and CD8^+^CXCR3^+^CD20^+^ cells; the median is shown for each donor. Differences were analysed using a Friedman test with Dunn’s multiple comparison test. The total number of cells analysed in the whole pancreatic slide per donor was 977,399 for E207B, 635,014 for E235, and 144,824 for E254. (**c**–**f**) Flow cytometric analysis of peripheral blood lymphocytes from ND donors (*n*=10) and adult RO donors with type 1 diabetes (*n*=10). (**c**) Representative plots showing CD8^+^CD20^+^ T cells. (**d**) Summary graph showing the frequency of CD8^+^CD20^+^ T cells. (**e**) Representative plots showing CXCR3 expression. (**f**) Summary graph showing the frequency of CXCR3^+^ cells in CD8^+^CD20^+^ and CD8^+^CD20^−^ T cells. Horizontal lines indicate the median. Differences were analysed using a two-way ANOVA with Tukey’s multiple comparison test. Statistically significant differences are indicated using asterisks: *p<0.05

On further examination of the CXCR3^+^CD8^+^ T cells identified in the pancreas of the young-onset donors, we observed that these T cells also had a distinct expression of CD20 (Fig. 6a). Using our analysis pipeline to distinguish the various T cell lineages in the pancreatic tissue, we subsequently evaluated the CD20 intensity on CD8^+^ cells only, CD20^+^ cells only and CD8^+^CXCR3^+^CD20^+^ cells. Analysis of the tissue demonstrated the level of CD20 expression on CD8^+^CXCR3^+^CD20^+^ B cells was dimmer, but not statistically different to CD20^+^ B cells only (*p*=0.15) (Fig. 6b). We further investigated these CD8^+^CD20^+^ T cells in the peripheral blood of RO donors by flow cytometry, and found no difference in the frequency of CD8^+^CD20^+^ T cells (gated on CD3^+^CD19^−^ cells, see ESM Fig. 4) between RO type 1 diabetes and ND donors (Fig. 6c, d). However, when we assessed the cells that co-expressed CXCR3, we observed an increased expression of CXCR3 in CD20^+^ T cells in the peripheral blood of RO donors (Fig. 6e, f). Together, our data show that, while CXCR3 is highly expressed in the pancreas of donors with RO type 1 diabetes who were diagnosed at <7 years of age, the expression is restricted to CD8^+^ T cells that co-express CD20. Moreover, we also show an increase in CXCR3 expression on CD20^+^CD8^+^ T cells in the peripheral blood of adult RO type 1 diabetes.

## Discussion

CXCR3 is a receptor that plays a key role in lymphocyte migration, and expression of its ligands CXCL9, CXCL10 and CXCL11 is associated with enhanced T cell trafficking to inflamed tissues. Similarly, CXCR3 mediates the attraction of IgG^+^ antibody-secreting cells into inflamed tissues [20], is indispensable for the recruitment of antibody-secreting cells into the inflamed central nervous system [24], and is induced by IFNγ via the transcription factor T-bet [25]. In this study, we demonstrate that the reduction of CXCR3 on antigen-experienced B cells (DN and SWM) is associated with the duration of disease, but, despite the reduction in CXCR3 expression ex vivo, B cells maintain their response to IFNγ such that they upregulate CXCR3 and produce CXCL10 and CXCL9 after stimulation, albeit to somewhat lesser extent than in individuals with a recent diagnosis. In donors with RO type 1 diabetes, B cells are more responsive in culture and readily upregulate CXCR3 and CD95, compared with ND individuals. Our data suggest that B cell responses to IFNγ are dynamic throughout disease progression, and that reduced CXCR3 expression in LD disease is not due to a state of exhaustion, despite B cells from RO individuals exhibiting a more robust CXCR3 response. A limitation of our study is that our culture conditions for total B cells consisted of IFNγ combined with either anti-IgM/anti-CD40 or TLR9 signal; and therefore some B cells may not have been stimulated by these conditions.

We studied three isoforms of CXCR3, which have mostly been studied in various cancers, with CXCR3A identified as a chemokine receptor, with its functional activities being modulated by the CXCR3B and CXCR3alt variants by the formation of various heterodimers [26]. However, we found no differences in isoform expression on memory B cells between individuals with type 1 diabetes and without diabetes, and we therefore conclude that the reduced expression of CXCR3 is also not due to an alteration in CXCR3 isoforms.

In pancreatic tissue from RO CD20^Hi^ individuals, we demonstrate that the CD20^+^ B cells identified in pancreatic inflammatory infiltrates and the surrounding islets did not express CXCR3, indicating that CXCR3 may not be involved in the migration of B cells through pancreatic tissue. It is also possible that CXCR3 was downregulated or cleaved upon arrival of the cells in the pancreas, and while we consider this unlikely due to the absolute absence of CXCR3 B cells across whole sections, we were not able to test this in a single snapshot in time. CXCR3-expressing B cells have been detected in the pancreatic lymph nodes of young-onset donors but pancreatic tissue was not investigated [23].

A limitation in our study is the sampling of only a small cohort. We investigated peripheral blood B cells in adults diagnosed with RO diabetes, but the pancreas samples were from individuals diagnosed at a young age. It should be noted that insulitis, and specifically CD20^+^ B cells, are up to 11-fold more prevalent in the islet infiltrate of individuals diagnosed at a young age [3, 4]. We show that antigen-experienced memory B cells in the circulation of donors with adult RO diabetes have comparable CXCR3 expression to that of ND donors, and that CXCR3 expression on pancreatic infiltrating B cells is lacking in young-onset individuals. Direct comparisons are required to fully understand whether there are differences in CXCR3 dynamics between individuals with young- vs adult-onset type 1 diabetes, to fully determine whether CXCR3 is required for B cells to migrate to the pancreas.

However, as fewer B cells are present in the pancreas in adult-onset type 1 diabetes, comparisons of CXCR3 expression in the pancreas of those who are older at diabetes onset with CXCR3 expression in young-onset disease may be challenging due to the limited number of cells available to study. Interestingly, in a study comparing atypical B cells between adults and children exposed to *Plasmodium falciparum*, it was demonstrated that CXCR3 expression was higher in adults compared with children, which was attributed to chronic antigen exposure [27]. Therefore, additional studies of circulating B cells in young- vs adult-onset diabetes may reveal important differential chemokine dynamics.

Our data suggest that a chemokine receptor other than CXCR3 may be responsible for the migration of B cells to the pancreas, with one possible candidate being the CXCR5/CXCL13 axis. CXCR5 is expressed on most B cells, although it is not expressed on DN2/atypical B cell subsets [28]. CXCL13 is a central chemokine in the recruitment of B cells and follicular T cells towards B cell follicles in secondary lymphoid organs [29]. CXCL13 is expressed in the inflamed pancreatic islets of a non-obese diabetic mouse model, and blockade of this pathway disrupted B cell structural organisation, although not pancreatic infiltrates [30]. Overall, our study shows that the reduced expression observed on antigen-experienced B cells in type 1 diabetes is not due to a failure to mount a response to IFNγ or different isoform expression. We speculate that the reduced expression is due to a ligand-induced internalisation in the B cells, initiated by the increase in ligands, such as CXCL10, observed during the progression of disease [14, 31], and our data indicate that CXCL10 stimulation reduces CXCR3 expression on memory B cells (ESM Fig. 3b). Another possibility is that CXCR3 is reduced due to the excess of cytokine production by both T and B cells during disease. IL-10 downregulates CXCR3 expression on CD4^+^ Th1 cells [32], and expression of this cytokine has been shown to be elevated in individuals with type 1 diabetes [33], specifically those who are older at onset [3]. Furthermore, a reduction in expression of the IFNγ receptor may also lead to a diminished B cell response, and lower CXCR3 expression.

CXCR3 expression has been detected on infiltrating islet CD3^+^ T cells in the pancreas of individuals with RO type 1 diabetes [17, 34], with both studies including adult-onset individuals. Most recently, it has been shown that CXCR3 gene expression on stem-like CD8 T cells in pancreatic lymph nodes is increased in autoantibody-positive individuals and those with type 1 diabetes [35]. In our studies, we observed that CXCR3 expression in the pancreas was restricted to a population of CD8^+^ T cells, which co-expressed similar levels of CD20 to those in CD20^+^ B cells in young-onset donors. Interestingly, not all CD8^+^ T cells were positive for CXCR3, and these CXCR3-negative CD8^+^ T cells were CD20-negative. CD20^+^ T cells have been identified in individuals with multiple sclerosis [36], and although CD20 expression is usually associated with B cells, its expression on T cells has been linked to activated memory T cells that produce proinflammatory cytokines such as IFNγ [37]. Such CD20^+^ T cells have been identified in multiple sclerosis lesions [38], and are enriched in the autoreactive myelin-specific CD8^+^ T cell population in individuals with multiple sclerosis [39]. CD20 expression on CD8^+^ T cells in the pancreas may indicate an interaction between T cells and B cells such that the CD20 molecule is associated with the CD8^+^ T cells via trogocytosis [40], the transfer from one cell to another. Furthermore, these CD20^+^ T cells, identified in cerebrospinal fluid in lesions from individuals with multiple sclerosis [41], have been attributed to a tissue-resident phenotype (T_RM_) and correlate with multiple sclerosis disease markers, and reportedly produce increased frequencies of proinflammatory cytokines, such as IFNγ [42]. However, it is possible that the subset of T cells that we have observed in our studies is distinct from these, and that the CD20 molecule is endogenously transcribed in this T cell population [43]. Further studies using spatial transcriptomics to allow measurement of RNA expression at the single-cell level may help to dissect this in the future.

In this small cohort, we observed no difference in the low frequency of CD8^+^CD20^+^ T cells in the peripheral blood of people with type 1 diabetes compared with non-diabetic donors. It should be noted that the CD20 intensity was distinctly lower in the peripheral blood compared with the pancreas, possibly suggesting greater B cell interaction with CXCR3^+^ T cells in the pancreatic tissue. We demonstrated an increase in CXCR3 expression on peripheral CD8^+^CD20^+^ T cells in RO individuals, suggesting that this subset of T cells exhibits enhanced migratory properties in adult individuals undergoing active beta cell destruction. However, whether this CD8^+^ T cell population with elevated CXCR3 expression is present or altered in those who are diagnosed at a young age is undetermined. To the best of our knowledge, this is the first combined study that describes CXCR3^+^CD20^+^ T cells in inflamed pancreatic tissue and the peripheral blood of individuals with type 1 diabetes, and future studies are needed to fully elucidate the role of these cells in the development of disease.

Overall, we show that the loss of CXCR3 expression is not due to a lack of response to proinflammatory cytokines, but is more likely due to a chemokine or cytokine feedback mechanism in antigen-experienced B cells that may be dependent on the stimuli to which the B cells have been exposed. Moreover, we demonstrate that a proportion of CXCR3^+^CD8^+^ T cells are also CD20^+^ in the pancreas of RO donors diagnosed at <7 years old, and these may represent an autoreactive, pathogenic subset with increased migratory properties. Accordingly, it may be important to consider the use of anti-CXCR3 and anti-CXCL10 treatments, both of which are now being used in the clinical setting for attenuating autoimmune disease [44, 45].

## Supplementary Information

Below is the link to the electronic supplementary material.

ESM 1 (PDF 3043 KB)

## Data Availability

Data are available from the corresponding authors upon reasonable request.

## Abbreviations

BCR: B cell receptor
CXCR3: C-X-C chemokine receptor type 3
DN: Double-negative (B cells)
EADB: Exeter Diabetes Archival Biobank
LD: Long-duration
ND: Non-diabetic
PBMC: Peripheral blood mononuclear cell
RO: Recent-onset
SWM: Switched memory B cell
TLR: Toll-like receptor
