# Supplementary material for: CXCR3 expression on antigen-experienced B cells is systemically dysregulated in type 1 diabetes
Source: Diabetologia. 2025 Nov 22;69(2):451–65. doi: 10.1007/s00125-025-06608-y (PMC12779670; doi:10.1007/s00125-025-06608-y)
Supplement: Supplementary file 1 — ESM 1 (PDF 3043 KB) [file 125_2025_6608_MOESM1_ESM.pdf]

ESM Methods

CXCR3 isoform assessment RNA concentrations were determined using the Nanodrop One (Thermo Scientific), and cDNA prepared using Applied Biosystems™ High-Capacity cDNA Reverse Transcription Kit, including RNase inhibitor, in a 20µl reaction containing ≤300ng RNA, as described by the manufacturer. Isoform mRNA expression was determined by qPCR using TaqMan™ Fast Advanced Master Mix for PCR, with standard cycling on a ViiA7 Real-Time PCR system (Applied Biosystems): CXCR3A forward primer 5'-CCCAGCAGCCAGAGCACC-3', reverse primer 5'-TCATAGGAAGAGCTGAAGTTCTCCA-3', probe 5'FAM-CATGGTCCTTGAGGTGAGTGACCACCAA-3'; CXCR3B forward primer 5'-TGCCAGGCCTTTACACAGC-3', reverse primer 5'-TCGGCGTCATTTAGCACTTG-3', probe 5'-FAM-CCCGTTCCCGCCCTCACAGG-3'as described [1, 2]. CXCR3alt forward primer 5'-CACGACGAGCGCCTCAA-3', reverse primer 5'-GTTGGGGCAGCCCAGG-3', probe 5'-FAM-CCGGAACTTGACCCCTGTGGGAAG-3' [2]. Primers and probes were obtained from Life Technologies, Sigma Aldrich and Invitrogen. Housekeeping gene expression was determined using Applied Biosytems TaqMan Pre-developed Assay Reagents with FAM-labelled probe/primer sets for Human ACTB and GAPDH. Samples were run in triplicate. Negative RT controls and H_2O blanks were included. To study the effect of CXCL10 on CXCR3 isoform expression, memory and naive B-cells isolated from fresh blood samples were incubated ±50nM recombinant human CXCL10 (BioLegend) in RPMI containing penicillin/streptomycin for 3 hours at 37°C, prior to RNA extraction. No difference in CXCR3 isoform expression between thawed and fresh samples were observed (data not shown).

Multiplex immunofluorescence staining Deparaffinisation was undertaken with Histoclear™; (SLS(UK); #NAT1334), rehydration in an alcohol series, and initial heat-induced epitope retrieval (HIER) (Citrate pH6) was extended to 20 minutes under pressure to address

archival fixation. Primary antibodies were applied in sequential rounds of the OPAL protocol (Akoya Manuals) to include an additional blocking step with peroxidase block (Dako) (5 minutes) prior to application of the Opal polymer HRP Ms + Rb reagent. Opal fluorophores where optimised as per manufacturers instruction (see ESM Table 2). Following completion of the six cycles of antigen visualisation, 4′,6-diamidino-2-phenylindole (DAPI, Invitrogen) was added at 1:1000 to the slides for 1 hour to facilitate subsequent cell detection algorithms. Sections were mounted with ProLong Diamond Antifade Mountant (ThermoFisher Scientific). Whole-section images were captured via the PhenoImagerHT Whole Slide Scanner (Akoya Biosciences, USA), and processed to remove spectral bleed through and autofluorescent signal using the native InForm software (v2.6). Whole slide image analysis was undertaken using QuPath (version 0.5.1)) [3]. Automatic cell detection was performed for whole tissue pancreas sections, and threshold classifiers were set for each fluorescent channel to identify positively-stained cells. Composite classifiers were generated for each cell phenotype of interest. Maximum fluorescent intensity (MFI) of CD20 for each cell phenotype was captured and all extracted datasets were processed in R (version 4.4.0).

[1] Ingelfinger F, Kuiper KL, Ulutekin C, et al. (2024) Twin study dissects CXCR3(+) memory B cells as non-heritable feature in multiple sclerosis. Med 5(4): 368-373 e363. 10.1016/j.medj.2024.02.013

[2] Reijm S, Kwekkeboom JC, Blomberg NJ, et al. (2023) Autoreactive B cells in rheumatoid arthritis include mainly activated CXCR3+ memory B cells and plasmablasts. JCI Insight 8(20). 10.1172/jci.insight.172006

[3] Bankhead P, Loughrey MB, Fernandez JA, et al. (2017) QuPath: Open source software for digital pathology image analysis. Sci Rep 7(1): 16878. 10.1038/s41598-017-17204-5

Type	Age, years	Sex	Mean age in group (years)	Age range in cohort (years)
Matched age and sex donors				
ND01	56	F	**41.6**	**21-69**
ND02	43	F		
ND03	50	F		
ND04	45	F		
ND05	59	M		
ND06	28	M		
ND07	32	M		
ND08	37	F		
ND09	39	M		
ND10	31	M		
ND11	25	F		
ND12	36	F		
ND13	24	F		
ND14	47	F		
ND15	34	M		
ND16	34	M		
ND17	49	M		
ND18	65	M		
ND19	57	F		
ND20	21	M		
ND21	40	F		
ND22	24	M		
ND23	31	M		
ND24	24	M		
ND25	58	M		
ND26	24	F		
ND27	29	M		
ND28	40	F		
ND29	55	M		
ND30	24	F		
ND31	65	M		
ND32	60	M		
ND33	49	F		
ND34	50	F		
ND35	28	M		
ND36	36	M		
ND37	50	F		
ND38	24	F		
ND38	69	M		
ND39	54	M		

ND40	43	F		
ND41	61	M		
ND42	69	M		
ND43	27	M		
ND44	54	M		
ND45	42	F		
ND46	38	M		
ND47	30	M		
ND48	56	M		
ND49	64	M		
ND50	27	F		
ND51	27	F		
ND52	23	F		
LD01	26	F	**43.3**	**21-68**
LD02	47	F		
LD03	51	M		
LD04	42	F		
LD05	57	F		
LD06	21	M		
LD07	61	M		
LD08	27	F		
LD09	21	F		
LD10	59	M		
LD11	59	M		
LD12	34	M		
LD13	45	F		
LD14	68	M		
LD15	52	M		
LD16	59	M		
LD17	41	F		
LD18	38	M		
LD19	30	M		
LD20	25	F		
LD21	68	M		
LD22	39	F		
LD23	31	F		
LD24	32	M		
LD25	52	F		
LD26	28	M		
LD27	57	M		
LD28	36	M		
LD29	66	M		
LD30	39	M		
LD31	54	M		
LD32	41	F		

LD33	64	F			
LD34	34	M			
LD35	27	F			
LD36	50	F			
LD37	23	F			
RO1	40	F	**37.5**	**20-64**	
RO2	34	M			
RO3	29	M			
RO4	24	M			
RO5	23	F			
RO6	31	M			
RO7	54	F			
RO8	57	M			
RO9	41	M			
RO10	39	M			
RO11	22	M			
RO12	53	M			
RO13	43	F			
RO14	22	M			
RO15	20	F			
RO16	53	F			
RO17	31	F			
RO18	25	M			
RO19	49	F			
RO20	47	M			
RO21	25	M			
RO22	64	F			
ND for CXCL10 stimulation studies					
ND53	26	F	**42.3**	**26-67**	
ND54	29	M			
ND55	42	F			
ND56	33	M			
ND57	54	M			
ND58	29	F			
ND59	59	M			
ND60	67	F			
EADB donor details					
Case	Age in years	Sex	Age at diagnosis	Disease duration	RRID
E207B	3	F	3	2 weeks	SAMN46311860
E235	6	M	6	Recent	SAMN46311856
E254	6	F	6	Recent	SAMN46311849

ESM Table 1. Peripheral blood and pancreas donor information.

Reagent	Source	Identifier
Flow Cytometry Antibodies		
Live Dead Aqua	Life Technologies	#L34957
Live Dead Blue	Life Technologies	#L23105
CD19, HIB19	BioLegend	#302229
CD20, 2H7	BioLegend	#302313
IgD, IA6-2	BioLegend	#348215
CD27, 0323	BioLegend	#302827
CD95, DX2	BioLegend	#305633
CXCR3, G025H7	BioLegend	#353705
CXCR3, 1C6	BD Biosciences	#561730
CD45, HI30	BD Biosciences	#563792
CD3, OKT3	BioLegend	#317332
CD20, 2H7	BioLegend	#302332
CD27, LG.3A10	StemCell	#60160PE.1
CD8, SK1	BioLegend	#344725
Human TruStain FcX	BioLegend	#422302
Immunohistochemistry Antibodies and kits		
Insulin	Invitrogen	14-9769-82
Glucagon	Abcam	ab10988
CD20	Dako	M0755
CD8	Dako	M7103
CXCR3	Abcam	ab52632
DAPI	Invitrogen	
Other Reagents		
CpG	Keck Oligonucleotide Facility, Yale School of Medicine	
hrCD40L	Biolegend	#591706
Anti-IgM	BioLegend	#314502
IFNγ	BioLegend	#570216

ESM Table 2. Reagents.

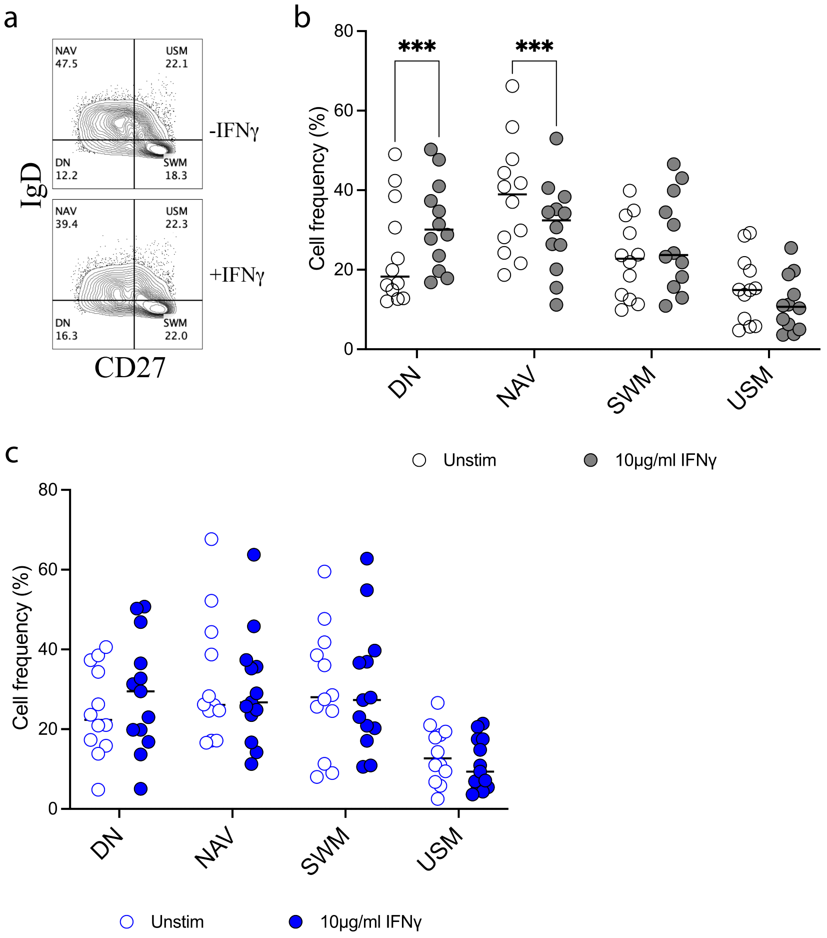

ESM Figure 1. B cell subset frequencies after treatment with IFNγ. Donors were assessed for CXCR3 responses to IFNγ. Isolated B-cells from PBMCs were cultured for 5 days before flow cytometric measurement and post-analysis gating was performed to identify double negative (DN), naive (NAV), switched memory (SWM) and unswitched memory (USM) B-cells (a). Frequencies for unstimulated (open circles) and IFNγ-stimulated (closed circles) populations after culture of B-cells from non-diabetic donors (a) and individuals with type 1 diabetes (b). *** p<0.001, two-way ANOVA with a Tukey's multiple comparison test.

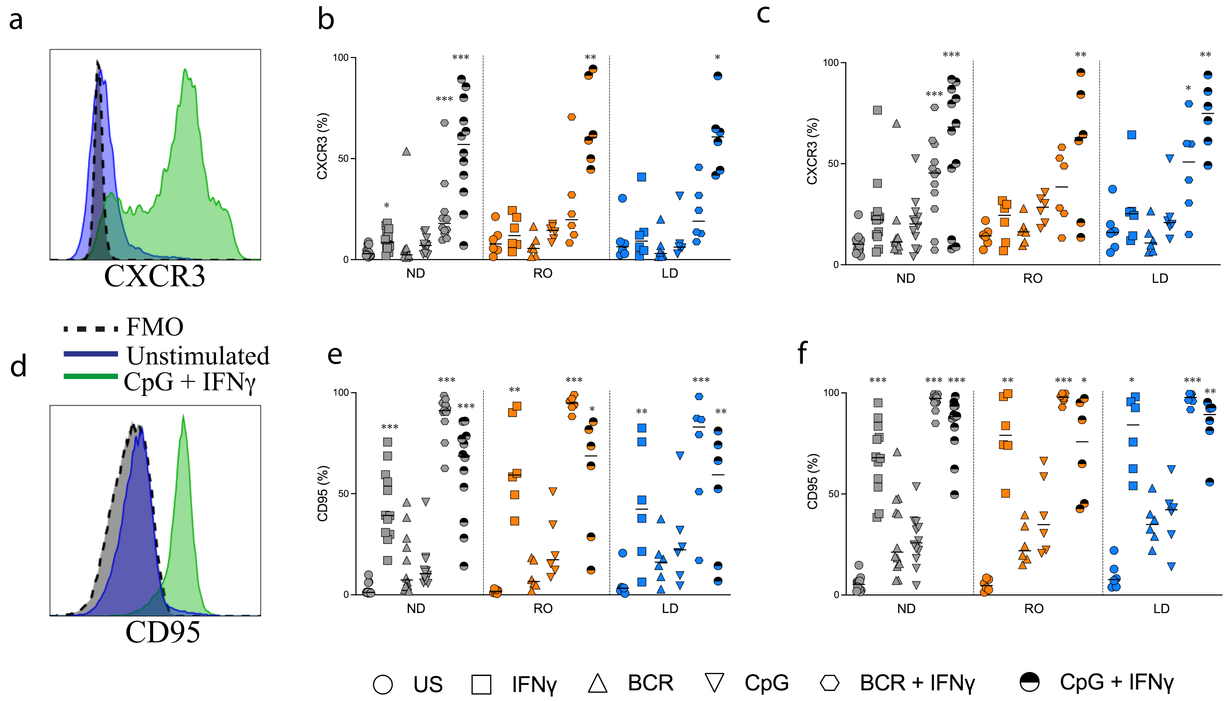

ESM Figure 2. Non diabetic (ND, *n* = 12), recent onset (RO, *n* = 6) and long duration (LD, *n* = 6) donors were assessed for a response to various stimuli. Isolated B-cells from PBMCs were cultured for 5 days before flow cytometric measurement and post-analysis gating was performed. (a) Representive fluorescence minus one controls (FMO) for CXCR3 (b, c) No differences in CXCR3 expression, shown as percentage, on (b) naive and (c) unswitched memory B-cells in recent onset or long duration individuals with various stimuli.
d) Representive fluorescence minus one controls (FMO) for CD95 (e, f) No differences in CD95 expression, shown as percentage, on (e) naive and (f) unswitched memory B-cells in recent onset or long duration individuals with various stimuli. Friedman test with a Dunn's multiple comparison test was performed between stimulations, all compared to unstimulated control, ** p<0.01, ** p<0.001. For statistical differences between cohorts a two-way ANOVA with a Tukey's multiple comparison test was performed, with no statistical differences observed. US; unstimulated, BCR; B Cell Receptor (anti-IgM & anti-CD40).

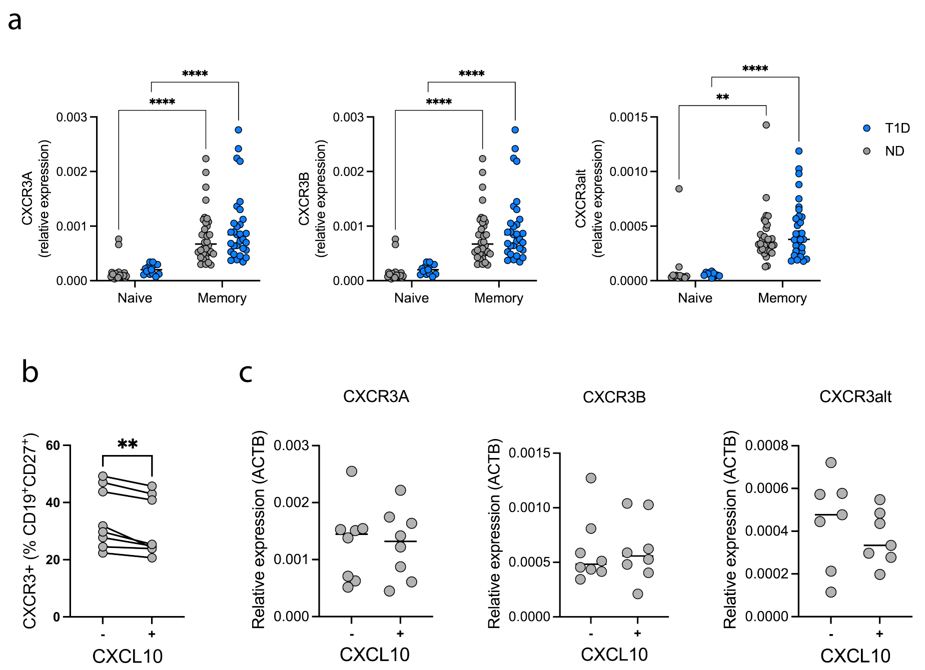

ESM Figure 3. CXCR3 isoforms on B-cells in type 1 diabetes a) Messenger RNA expression of CXCR3A, B and alt isoforms in naïve and memory B-cells from non-diabetic (ND) and individuals with type 1 diabetes (T1D), compared against actin B. Median values were compared between the control and diabetes groups. A two-way ANOVA was performed. Within study groups there were highly significant differences between naive and memory B cells for all isoforms (p<0.01). b) Summary graph demonstrating CXCR3 expression between CXCL10 stimulated and unstimulated memory B cells from non-diabetic controls. A Wilcoxon test was performed. c) Messenger RNA expression of CXCR3A, B and alt isoforms in unstimulated and CXCL10 stimulated memory B cells from ND donors, compared against actin B. Median values were compared and Mann Whitney tests were performed.

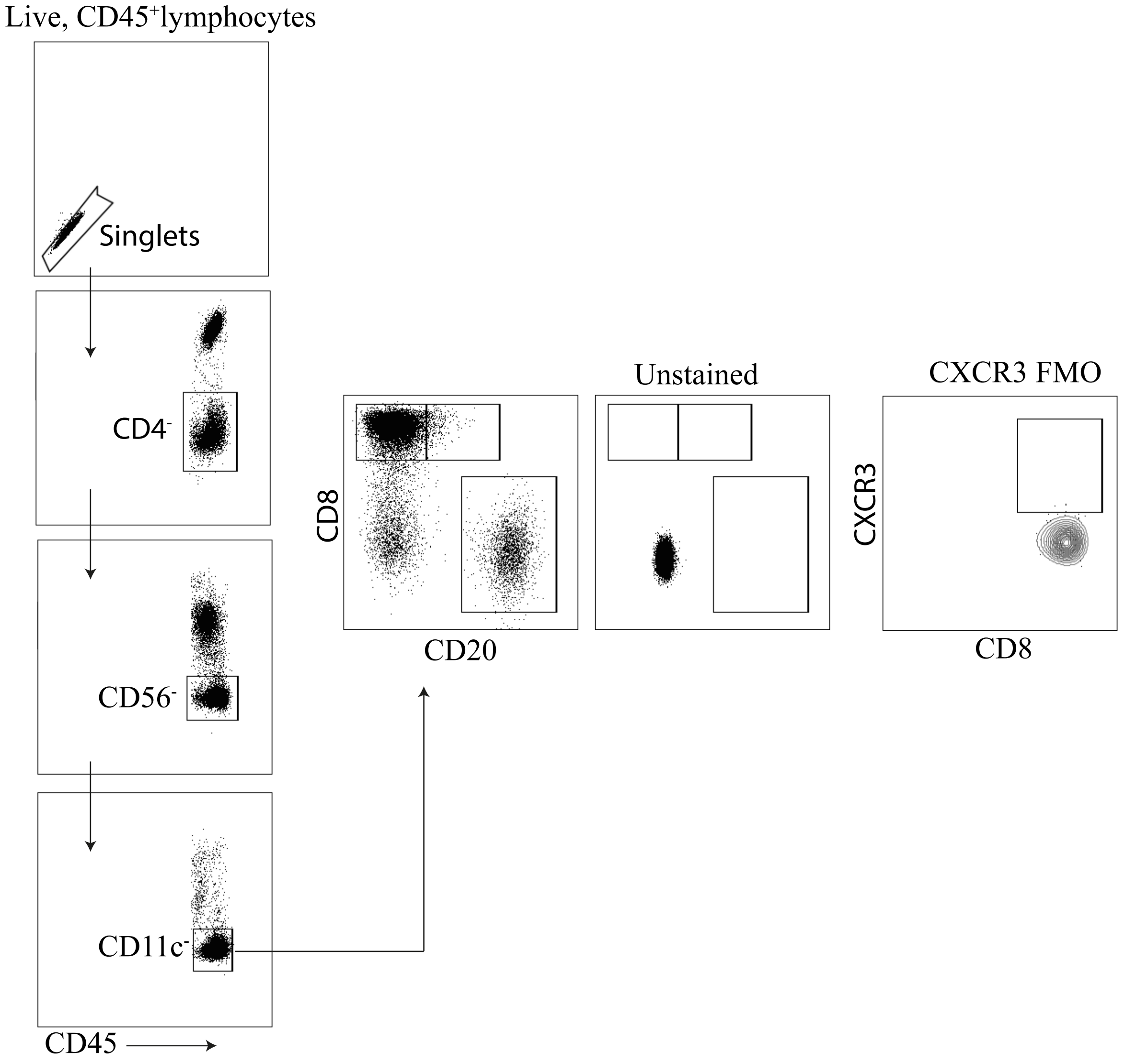

ESM Figure 4. Gating strategy for CD8⁺CD20⁺ T cells. Live CD45⁺ lymphocytes were selected for singlets, and gated on CD4⁻, CD56⁻, CD11c⁻ CD8⁺ T cells. Plots show unstained and CXCR3 fluorescence minus one (FMO).
